# Coordinated orphan disease research: yes, we can!

**DOI:** 10.3389/fgene.2013.00207

**Published:** 2013-11-01

**Authors:** Olivier M. Vanakker

**Affiliations:** Center for Medical Genetics, Ghent University HospitalGhent, Belgium

**Keywords:** pseudoxanthoma elasticum, ectopic mineralization, ABCC6, orphan disease, connective tissue diseases, ENPP1

Research in the field of orphan diseases is confronted with several hurdles. Not only lack of knowledge and insufficient awareness for these disorders among the general public, politicians and yes, often also among the medical and scientific community, but also limited funding, dispersed research initiatives and seemingly unrelated datasets usually make it more difficult to progress in the understanding of these disorders. Ectopic mineralization diseases, with pseudoxanthoma elasticum (PXE) as a paradigm disorder, have been no exception to the rule in facing all of these hurdles. But at the same time, PXE has been a prime example of the efforts to overcome these barriers. And to a certain point, they can be overcome as this Special Topic of Frontiers in Systems Biology proves.

Soft tissue mineralization is the result of a delicate interplay of a large number of protagonists—transcription factors, calcification promoting and inhibiting proteins and enzymes, homeostatic cues (Schinke et al., 1999). It is precisely this complexity that makes soft tissue calcification so difficult to comprehend. At the same time, it is the prototype of a pathophysiological mechanism that cries out for a concerted action of different fields of expertise to build our insights into how it functions. Indeed, there are many faces to soft tissue mineralization, several of which are captured in PXE. This rare autosomal recessive connective tissue disorder is characterized by ocular, skin, and cardiovascular symptoms and can present important variability, particularly in the severity of symptoms (Vanakker et al., 2008). The histological hallmark of PXE is mineralization and fragmentation of elastic fibers. PXE was shown to be caused by mutations in the *ABCC6* gene, encoding an ATP-dependent transporter protein, though the physiological role and pathological consequences of ABCC6 remain unclear (Le Saux et al., 2001).

To achieve the ultimate goal of our research, a treatment to stabilize or cure PXE, there is a need to understand the function of the ABCC6 protein which is perturbed in PXE. Despite the considerable progress made in the past years, which is reflected on in this issue, many crucial questions remain unanswered. Concerted action also implies that we can look beyond our own protein of interest to other related proteins and diseases; their story—the difficulties as much as the successes—may give us valuable insights in how we can proceed in understanding our ABC-transporter of interest.

Next to mechanistic insights, the phenotype of patients should be defined in the greatest detail. The importance of this for the follow-up of patients goes without saying but cannot be seen independently of our quest to understand the mechanisms of disease. In this respect, recent findings on the unique characteristics of the PXE vasculopathy and advances in the ophthalmological features of PXE can give us important clues for the mechanisms that lie beneath (De Zaeytijd et al., 2010; Lefthériotis et al., 2011; Campens et al., 2013).

Finally, the molecular biology of these disorders needs be further refined. More than a decade after the identification of *ABCC6* as the causal gene for PXE, the molecular basis of PXE has become increasingly more complex then was initially conceived. Not only have a number of genes been identified which are associated with similar phenotypes, such as *GGCX* or *ENPP1*, but the variability of the PXE phenotype has also triggered the search for modifier genes (Vanakker et al., 2007; Nitschke et al., 2012). Though until now most of the secondary genes with a link to the phenotype should be considered susceptibility factors in stead of true modifiers, prudent progress has been made through the characterization of the role of *VEGF-A* variants in ocular neovascularization (Zarbock et al., 2009).

The drive behind research endeavors aiming to understand a group of rare disorders is the result of the enthusiasm of a group of dedicated scientists but—no less—also of patients and their representing organizations. Despite the use of animal models which can be very valuable for certain aspects of disease, the active involvement of patients in research, comprising clinical studies and experiments on tissues, remains invaluable for the future of PXE research. The advocacy groups of these patients play an important role in translating research findings to the patients but have often also been a thriving force to gather and stimulate researchers and physicians around the world.

In this special topic of Frontiers in Systems Biology, some of the leading scientists and physicians in the field of PXE and ectopic mineralization disorders have summarized the current knowledge in their particular field of expertise and share their thoughts on how further progress can be made. Basic science contributions cover the complexity of ABCC6 transporter function in (patho)physiological mineralization, the role of mesenchymal cells such as fibroblasts and mechanisms of remodeling in calcified vasculopathies. A perspective on the relevance of other ABC transporters for the study of ABCC6 and a methodological review on one of the innovative model organisms, the *Danio Rerio* or zebrafish, provide a balance between current and future endeavors. The molecular etiology is covered by reviews on the transcriptional regulation of the *ABCC6* gene and the role of modifier genes in PXE, while a contribution on *ENPP1* and the overlapping phenotype between PXE and Generalized Arterial Calcification of Infancy (GACI) makes the transition to clinical papers on specific aspects of the PXE retinopathy and vasculopathy. This research topic concludes with a perspective on the current and future role of advocacy groups in orphan disease research.

The rarity of hereditary soft tissue mineralization disorders require a coordinated effort at all levels mentioned above to obtain faster advances in knowledge of disease pathophysiology and its rapid translation into the clinic. This will inevitably improve health care for patients suffering from these orphan diseases, but may also lead to valuable insights on more common disorders such as atherosclerosis and stroke as well as on the process of aging. Though maybe not always obvious at first sight, there is thus, unquestionably a relation with some of the global health care problems the world is facing today and will continue to face in the future. This Special Topic evidences how the work of several research groups on rare mineralization diseases can be brought together to reflect what is currently living in the field. At the same time, it should be an incentive to work further on a platform to integrate the research findings in these diseases retro- and prospectively. Only by uniting our attempts to gain insights in the complexity of soft tissue mineralization will we be able to get a grip on these diseases.

## References

[B1] Campens L. Vanakker O. M. Trachet B. Segers P. Leroy B. P. De Zaeytijd J. (2013). Characterization of cardiovascular involvement in pseudoxanthoma elasticum families. Ather. Thromb. Vasc. Biol. 33, 2646–2652 10.1161/ATVBAHA.113.301901 23968982

[B2] De Zaeytijd J. Vanakker O. M. Coucke P. J. De Paepe A. De Laey J. J Leroy B. P. (2010). Added value of infrared, red-free and autofluorescence funcus imaging in pseudoxanthoma elasticum. Br. J. Ophthalmol. 94, 479–486 10.1136/bjo.2009.162644 19726431

[B3] Lefthériotis G. Abraham P. Le Corre Y. Le Saux O. Henrion D. Ducluzeau P. H. (2011). Relationship between ankle brachial index and arterial remodeling in pseudoxanthoma elasticum. J. Vasc. Surg. 54, 1390–1394 10.1016/j.jvs.2011.04.041 21723076 PMC5529101

[B4] Le Saux O. Beck K. Sachsinger C. Silvestri C. Treiber C. Göring H. H. (2001). A spectrum of ABCC6 mutations is responsible for pseudoxanthoma elasticum. Am. J. Hum. Genet. 69, 749–764 10.1086/323704 11536079 PMC1226061

[B5] Nitschke Y. Baujat G. Botschen U. Wittkampf T. du Moulin M. Stella J. (2012). Generalized arterial calcification of infancy and pseudoxanthoma elasticum can be caused by mutations in either ENPP1 or ABCC6. Am. J. Hum. Genet. 90, 25–39 10.1016/j.ajhg.2011.11.020 22209248 PMC3257960

[B6] Schinke T. McKee M. D. Karsenty G. (1999). Extracellular matrix calcification: where is the action? Nat. Genet. 21, 225–229 9988260 10.1038/5928

[B7] Vanakker O. M. Leroy B. P. Coucke P. Bercovitch L. G. Uitto J. Viljoen D. (2008). Novel clinico-molecular insights in pseudoxanthoma elasticum provide an efficient molecular screening method and a comprehensive diagnostic flowchart. Hum. Mut. 29:205 10.1002/humu.9514 18157818

[B8] Vanakker O. M. Martin L. Gheduzzi D. Leroy B. P. Loeys B. L. Guerci V. I. (2007). Pseudoxanthoma elasticum-like phenotype with cutis laxa and multiple coagulation factor deficiency represents a separate genetic entity. J. Invest. Dermatol. 127, 581–587 10.1038/sj.jid.5700610 17110937

[B9] Zarbock R. Hendig D. Szlsika C. Kleesiek K. Gotting C. (2009). Vascular endothelial growth facyor gene polymorphisms as prognostic markers for ocular manifestations in pseudoxanthoma elasticum. Hum. Mol. Genet. 18, 3344–3351 10.1093/hmg/ddp259 19483196

